# Recombinant BCG With Bacterial Signaling Molecule Cyclic di-AMP as Endogenous Adjuvant Induces Elevated Immune Responses After *Mycobacterium tuberculosis* Infection

**DOI:** 10.3389/fimmu.2019.01519

**Published:** 2019-07-03

**Authors:** Huanhuan Ning, Lifei Wang, Jie Zhou, Yanzhi Lu, Jian Kang, Tianbing Ding, Lixin Shen, Zhikai Xu, Yinlan Bai

**Affiliations:** ^1^Department of Microbiology and Pathogen Biology, Air Force Medical University, Xi'an, China; ^2^Key Laboratory of Resources Biology and Biotechnology in Western China, Ministry of Education, College of Life Sciences, Northwest University, Xi'an, China; ^3^Department of Endocrinology, Xijing Hospital, Air Force Medical University, Xi'an, China; ^4^College of Medicine, Xijing University, Xi'an, China

**Keywords:** *Mycobacterium tuberculosis*, recombinant BCG, cyclic di-AMP, adjuvant, immune response

## Abstract

Bacillus Calmette-Guerin (BCG) is a live attenuated vaccine against tuberculosis (TB) and remains the most commonly used vaccine worldwide. However, BCG has varied protective efficiency in adults and has safety concerns in immunocompromised population. Thus, effective vaccines are necessary for preventing the prevalence of TB. Cyclic di-AMP (c-di-AMP) is a bacterial second messenger which regulates various cellular processes and host immune response. Previous work found that c-di-AMP regulates bacterial physiological function, pathogenicity and host type I IFN response. In this study, we constructed a recombinant BCG (rBCG) by overexpressing DisA, the diadenylate cyclase of *Mycobacterium tuberculosis* (Mtb), and observed the physiological changes of rBCG-DisA. The immunological characteristics of rBCG-DisA were investigated on humoral and cellar immune responses in a mice infection model. Our study demonstrated that overexpression of DisA in BCG does not affect the growth but reduces the length of BCG. rBCG-DisA-immunized mice show similar humoral and cellar immune responses in BCG-immunized mice. After Mtb infection, the splenic lymphocytes from both BCG and rBCG-DisA-immunized mice produced more IFN-γ, IL-2, and IL-10 than the un-immunized (UN) mice, while the cytokine levels of the rBCG-DisA group increased significantly than those of the BCG group. The transcription of IFN-β, IL-1β and autophagy related genes (Atgs) were up-regulated in macrophages after treated with c-di-AMP or bacterial infection. The productions of IL-6 were increased after Mtb challenge, especially in the rBCG-DisA-immunized mice. Strikingly, H3K4me3, the epigenetic marker of innate immune memory, was found in both two immunized groups, and the rBCG-DisA group showed stronger expression of H3K4me3 than that of BCG. In addition, the pathological changes of rBCG-DisA immunized mice were similar to that of BCG-immunized mice. The bacterial burdens in the lungs and spleens of BCG- and rBCG-DisA-immunized mice were significantly decreased, but there was no significant difference between the two immunized groups. Together, these results suggested that compared to BCG, rBCG-DisA vaccination, induces stronger immune responses but did not provided additional protection against Mtb infection in this study, which may be related to the innate immunity memory. Hence, c-di-AMP is a promising immunomodulator for a further developed BCG as a better vaccine.

## Introduction

Tuberculosis (TB), caused by *Mycobacterium tuberculosis* (Mtb), remains one of the most deadly killers of infectious diseases worldwide (1). The only vaccine currently available for TB is *Mycobacterium bovis* Bacillus Calmette-Guerin (BCG), which can effectively prevent severe TB in children but is less successful in controlling pulmonary TB in adults, and may cause disseminated infection in immunocompromised populations when it is administered as a live attenuated vaccine. By now 12 new vaccine candidates had been investigated in the clinical trials, including subunit vaccines, DNA vaccines, auxotrophic vaccines, and recombinant BCG (rBCG) (1). But so far, vaccines that can replace traditional BCG have not yet been obtained.

In addition to BCG's effects on TB, it has been shown that BCG also induces heterologous protections against non-mycobacterial reinfection, allergic diseases, and certain malignancies (2). Mounting evidence has been accumulated that these protection effects are mediated partly by an innate immune response, termed “innate immune memory” or “trained immunity,” which is mediated by epigenetic, metabolic, and functional reprogramming of innate immune cells (2–4). The process of trained immunity may also play a role in the beneficial effects of BCG against tuberculosis (2). Thus more attempts should be done to improve the safety and protection efficiency of BCG against TB.

Bacterial cyclic nucleotides have been proved to regulate various cellular processes as signal molecules (5, 6), including Cyclic AMP, (p)ppGpp (7–9), Cyclic di-GMP (c-di-GMP) (10), Cyclic di-AMP (c-di-AMP) and cGAMP (2′,3′ and 3′,3′) (11– 13). c-di-AMP was first identified by Witte in *Bacillus subtilis* and *Thermotoga maritime* in 2008 (14), which was synthesized from two ATP molecules by DNA integrity scanning protein (DisA), the first diadenylate cyclase (DAC). In bacteria c-di-AMP is hydrolyzed by phosphodiesterase (PDE) into AMP finally (15). The genes encoding DAC and PDE enzymes were found in a variety of bacteria, including *Listeria monocytogenes* (16), *Staphylococcus aureus* (17), *Streptococcus pneumoniae* (18), *Streptococcus pyogenes* (19), Mtb (20), and *Mycobacterium smegmatis* (Ms) (21). By now, it is found that c-di-AMP regulates different physiological processes in bacteria, including cell wall homeostasis (17), fatty acid metabolism (22), bacterial growth (15), spore formation (23), biofilm formation (24), and virulence (25). Moreover, c-di-AMP sensed by the innate immune system in the host cytosol leading primarily to the induction of type I interferon (IFN) via a STING-cGAS signaling axis (26, 27), while being also entangled in the activation of the NF-κB pathway (28, 29). In addition, the NLRP3 inflammasome is activated, either directly or indirectly by c-di-AMP, leading to IL-1β production (30).

Other than this, c-di-AMP as mucosal adjuvant with β-galactosidase could induce specific IgG and sIgA, and Th1/Th2/Th17 responses in mice model (31). Several studies proved that genetic manipulation of DAC or PDE or environmental stimulation could disrupt the homeostatic balance of c-di-AMP in bacteria (15, 16, 25, 32, 33). In conclusion, c-di-AMP regulates bacterial physiology including pathogenicity, as well as the host immune responses, which means its potential use in vaccines.

Our previous work initially confirmed that Rv3586 (DacA, or DisA) is the only Dac in Mtb, which is the ortholog of *B. subtilis* DisA (18). Further we proved that Rv2837c is the second cyclic nucleotide phosphodiesterase (CnpB) as a c-di-AMP hydrolyzing enzyme (15). It has been proved that c-di-AMP involves in the regulation of cell size and aggregation, virulence, and RNA abundance in mycobacterium. Previously study found that deletion of CnpB or overexpression of DisA significantly enhanced the c-di-AMP accumulation in Mtb, BCG, as well as Ms (15, 22, 25, 34). And elevated c-di-AMP levels in Mtb resulted in significant virulence attenuation in a mouse TB model (15, 25, 33). Notably, it has also been shown that c-di-AMP from *cnpB* deleted or *disA* overexpressed Mtb strains activate type I IFN response, autophagy, and limits the growth of bacteria within infected cells (15, 25). Additionally, c-di-AMP alone exerts immune stimulatory effects on antigen presenting cells including dendritic cells and macrophages both *in vitro* and *in vivo* (35). Therefore, it is speculated that c-di-AMP could act as an endogenous adjuvant to activate host immune response, as well as live vaccine virulence.

In this study, we found that DisA exhibited strong immunogenicity but the expression levels of DisA in Mtb and BCG were fairly low (36). We also noticed that the levels of DisA-specific antibodies in serum of TB patients and Mtb-infected mice were very low. Therefore, we constructed a recombinant BCG with elevated c-di-AMP by overexpressing *disA* (rBCG-DisA), determined its biological and immunological properties and investigated the immune enhancement by c-di-AMP as an endogenous adjuvant during vaccination with the rBCG.

## Materials and Methods

### Ethics Statement

The animal studies were conducted under the approval of Institutional Ethics Committee of Second Affiliated Hospital of Air Force Medical University, using the recommendations from the Guide for the Care and Use of Laboratory Animals of the Institute (approval no. TDLL-2016325).

### Bacterial Strains, Plasmids, Cell Lines, and Animals

*Escherichia coli* strain DH5α was used for mycobacteria-*E. coli* shuttle vector construction. *E. coli* DH5α was grown in Luria-Bertani broth or on Luria-Bertani agar plates. *Mycobacterium bovis* BCG (BCG Pasteur, ATCC 35734) and Mtb H37Ra (National Institutes for Food and Drug Control, China) were used in animal study. Mycobacteria strains were grown in Middlebrook 7H9 medium (BD) supplemented with 0.5% glycerol, 10% oleic acid albumin-dextrose-catalase (OADC) (BD), 0.05% Tween-80 (Sigma), or on 7H10 medium (BD) plates supplemented with 10% OADC at 37°C. Murine macrophage cells RAW264.7 was used for *in vitro* experiments. Female BALB/c mice from Animal Center of Air Force Medical University, aged 6–8 weeks, were used for studies on the immune responses and protection efficacy.

### BCG Protein Preparation

BCG was grown in 100 mL 7H9 medium supplemented with 10% OADC until OD_600_ ≈ 1.0. Bacteria were harvested, washed with lysis buffer (0.5% SDS, 50 mM Tris-HCl, 10 mM EDTA) for once and PBS for twice. Cells were resuspended in 8 mL cool PBS containing proteinase inhibitor cocktail (Roche) followed by sonication on ice. The lysate was centrifuged 12 000 rpm for 20 min at 4°C. Supernatant were transferred and stored at −80°C. The concentration of BCG proteins was detected by BCA protein assay.

### Overexpression of *disA* in BCG

The *disA* gene of Mtb, Rv3586, was PCR-amplified from Mtb H37Rv chromosomal DNA using gene-specific primers (Table S1). The amplicons were cloned into PW-54, an *E. coli*-mycobacterium shuttle vector, at the *Hin*d III restriction sites. The resulting construct PW54-*disA* was sequenced and subsequently transformed into BCG by electroporation. The recombinant BCG clones was selected on Middlebrook 7H10 medium plates supplemented with 10% OADC and 25 μg/mL kanamycin, and confirmed by colony PCR using plasmid specific primers and *disA* reverse primers (Table S1). Then the positive rBCG clone was inoculated in Middlebrook 7H9 medium supplemented with 0.5% glycerol, 10% OADC, 0.05% Tween-80 for expanding cultivation. Overexpression of *disA* in the recombinant BCG was further confirmed by Western blot with an anti-DisA polyclonal antibody (36).

### Determination of c-di-AMP Levels in Bacteria by HPLC

The c-di-AMP levels of bacterial strains were measured by high performance liquid chromatography (HPLC). BCG and rBCG strains were grown to OD_600_≈1.0 in Middlebrook 7H9 with 10% OADC and harvested by centrifugation. The bacteria were resuspended in extraction buffer containing acetonitrile (Sigma), methanol (Sigma), and water (2:2:1). The suspension was incubated on ice for 15 min, followed by boiling at 95°C for 10 min and then cooled down on ice. The lysates were centrifuged for 10 min at 4°C at 20 800 × *g* and then transferred into fresh tubes. The extraction was repeated twice. The pooled extractions were vacuum dried and dissolved in sterile water. Ten microliter of each sample was injected and separated by reverse-phase HPLC with a C-18 column (150 × 2.0 mm column) (Phenomenex) using a Waters 2695 Separations Module. Nucleotides were monitored at 254 nm (25, 37). Purified c-di-AMP (InvivoGen) was used to generate the standard curve. Intracellular c-di-AMP level was normalized by the corresponding bacterial wet weight.

### Determination of Bacterial Growth and Size

BCG and rBCG were inoculated in 7H9 with 10% OADC and the growth was determined by measuring OD_600_ at daily for 2 weeks. The growth curves were obtained after triplicate experiments. When BCG and rBCG strains were, respectively, grown in late-log phase, the bacteria samples were smeared onto glass slides and stained with Ziehl-Neelsen staining for acid-fast bacteria (15), while the rest of bacteria were washed with PBS twice and resuspended in PBS. Twenty microliter of the bacterial suspension was dropped onto the 200 mesh copper net, and excess bacterial suspension was absorbed by filter paper after 10 min incubation in the fume hood. The copper net was stained by phosphotungstic acid, and the bacterial morphology was observed by transmission electron microscopy (TEM) (TECNAI G2 Spirit Biotwin). The bacterial length was analyzed by Image *J* software with 100 bacterial images randomly selected in 10 observation fields.

### Infection of Macrophages

RAW264.7 cells were cultured in RPMI medium (Hyclone) with 10% heat-inactivated FBS (Sijiqing, China). Infections were carried out in 6-well plates in triplicate. For infection, early log-phase cultures of BCG and rBCG were washed and diluted in antibiotic-free RPMI and were added to the cells with a multiplicity of infection (MOI) at 10:1. The infection was allowed to continue for 4 h. Then the extracellular bacteria were removed by washing the infected cells with sterile PBS thoroughly. At 16 h after infection, macrophage cells were washed thoroughly with PBS followed, respectively, by lysates in RIPA buffer (TIANGEN, China) for Western blot and in TRIZol for RNA extraction.

### BCG Vaccination

Mice were anesthetized with an intraperitoneal injection of 50 mg/kg pentobarbital sodium. Then 10^6^ CFU of BCG and rBCG was injected subcutaneously in a volume of 100 μL PBS into the right back. PBS was injected as un-vaccinated/naive control.

### Splenocytes Proliferation Assay

Spleens from all groups were separated and squeezed through a 40 μm mesh strainer in 4 mL RPMI medium supplemented with 10% fetal bovine serum and 100 U/mL penicillin/0.1 mg/mL streptomycin (Shenggong, China). Cells were collected at 1 000 rpm for 5 min and then resuspended in 5 mL fresh red blood cell lysis buffer (Solarbio, USA) for 2 min, followed by addition of completed RPMI medium. After washing with fresh medium, 1 × 10^6^ splenocytes were seeded in 96-well microplates with 25 μg/mL BCG proteins and incubated at 37°C for 72 h. Each well was added with 20 μL MTS (3-(4,5-dimethylthiazol-2-yl)-5-(3-carboxymethoxyphenyl)-2-(4-sulfophenyl)- 2H-tetrazolium) (Promega, USA) and incubated for another 4 h, then measured at the absorbance of 490 nm (A_490_). Stimulation index (SI) = (A_490_ of stimulated wells—A_490_ of blank wells)/(A_490_ of negative wells—A_490_ of blank wells) (38).

CFSE (5(6)-Carboxyfluorescein diacetate N-succinimidyl ester) (Sigma, USA) assay was performed similarly to our previous study (38). Briefly, 1 × 10^6^ splenocytes were co-incubated with 5 μM CFSE at 37°C for 20 min. Subsequently, four volumes of cold RPMI 1640 containing 10% FBS were added to stop the reaction. After cells were washed and resuspended, cells were seeded in 12-well plates and stimulated with 25 μg/mL BCG proteins. Un-stimulated CFSE stain cells were regarded as non-proliferated cells. After 7-day incubation, cells were collected for flow cytometry analysis on 488 nm excitation source. Data were analyzed by Modfit 5.0 software and proliferation index = total cell numbers after proliferation/total cell numbers before proliferation (38).

### Flow Cytometry

Splenocytes were prepared from mice as described above. 1 × 10^6^ splenocytes were resuspended in 100 μL staining buffer (BioLegend, USA) and incubated with anti-CD32/16 mAbs (BioLegend) for 10 min. Cells were washed and incubated in 100 μL staining buffer with surface antibodies for 30 min on ice in dark, then washed with staining buffer. Cells were fixed in formalin for 10 min at room temperature and washed with staining buffer. Finally, cells were resuspended in 500 μL staining buffer for flow cytometry.

Intracellular cytokine labeling was performed after surface molecule staining as described above. Splenocytes were seeded in 96-well plates, stimulated with BCG protein (25 μg/mL) and then incubated at 37°C for 12 h. Brefeldin A (BioLegend, USA) was added into wells for another 12 h of incubation at the final concentration of 5 μg/mL. Cells were then washed and labeled with cell surface antibodies as described above. Then fixed cells were permeated with 1 mL Fix/Perm buffer (BD, USA) for 20 min. Cells were washed with 1 mL 1 × Perm/Wash buffer (BD, USA) twice, then pelleted and resuspended in 100 μL 1 × Perm/Wash buffer. Cells were incubated with titrated intracellular antibodies for 30 min at room temperature. After washing thrice, cells were resuspended in volumes of 500 μL staining buffer for flow cytometry.

### qRT-PCR

Total RNA was extracted from cells using Trizol reagent (Invitrogen, USA), according to the manufacturer's instructions. Five hundred nanogram of total RNA was reverse transcribed using the 5 × RT Mastermix (Takara, Japan). Expressions of cytokines were determined by qPCR using SYRB Green (Takara, Japan). The qRT-PCR using primers listed in Table S1. Gene expression data are presented as relative expression to GAPDH.

### ELISA

Sera from four mice in each group were assayed by ELISA with BCG protein (10 μg/mL) as coating antigen, and HRP-conjugated Goat Anti-Mouse IgG (Abcam, UK) for detection. *In vitro* splenocytes culture supernatants were collected and used for measurement of IFN-γ, IL-2, IL-10 and IL-6, respectively, by the ELISA per the manufacture's manuals (eBioscience, USA).

### Histopathology and Immunohistochemistry

Upper lobes of left lungs were fixed in 10% buffered formalin and sections of 5 μm in thickness from formalin fixed and paraffin embedded tissues were cut onto glass slides. H&E staining for pathohistological analysis was performed by the Department of Histopathology (Air Force Medical University, China). H3K4me3 expression was determined by immunohistochemistry (IHC), and IHC was performed by Shenyang Wanleibio Biotechnology Limited Company (China). Antibodies used in IHC were polyclonal rabbit anti-H3-K4 trimethyl (1:500, Abcam) and horseradish peroxidase-conjugated anti-rabbit antibody (1:500, Jackson Immunoresearch Laboratories).

### Infection of Mice and CFU Enumeration

Four weeks after the final immunization, mice were challenged intravenously (i.v.) by tail injection with 5 × 10^4^ CFU Mtb H37Rv in 100 μL of PBS. Eight weeks after Mtb challenge, lungs, and spleens were removed and homogenized in 5 mL of PBS using a 40 μm mesh strainer. Serial dilutions of organ homogenates were spread on 7H10 agar plates (+OADC supplement) for CFU counting after 3-week of incubation at 37°C, as described before (15).

### Statistical Analysis

Statistical analyses were performed using Graphpad Prism 5 Software (Graphpad Software, USA). Statistical significance was determined by Student *t*-test or a one-way ANOVA followed by Tukey post-test. Only *P*-values < 0.05 were considered statistically significant.

## Results

### The Expression of *disA* in Mycobacteria Was Low and the Specific Anti-DisA Antibodies in TB Patients and Mtb-Infected Mice Were Low as Well

**Figure 1:**
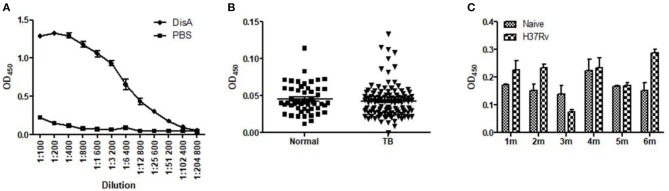
Specific anti-DisA antibodies in DisA immunized mice, TB patients, and Mtb-infected mice. **(A)** Sera were collected from purified protein DisA immunized BALB/c mice in our previous study and sera from PBS immunized mice as control, antibody titers against DisA were assayed by ELISA as shown dilution. **(B)** Antibody titers against DisA in TB patients (*n* = 53) and healthy control (*n* = 113) were assayed by ELISA (1:200). **(C)** Antibody titers against DisA in Mtb-infected mice. Sera were collected from BALB/c mice monthly at 1–6 month (m) post-infection, and specific anti-DisA antibody titers were detected by ELISA (1:200).

In previous work, we found that recombinant DisA protein induced high humoral immune response in mice, which indicates that DisA has strong immunogenicity (36). Anti-DisA antibody titers reached to 1:256 00 in mice sera after three times immunization with purified DisA (Figure 1A). However, DisA expressions were very low in Mtb and BCG detected by anti-DisA sera (36), which were consisted with the low level of c-di-AMP in bacteria (15, 25). As expected, the levels of DisA-specific antibodies in sera of TB patients and Mtb-infected mice were relatively low (Figures 1B,C).

### Expression of *disA* in BCG Elevates Bacterial c-di-AMP Levels

**Figure 2:**
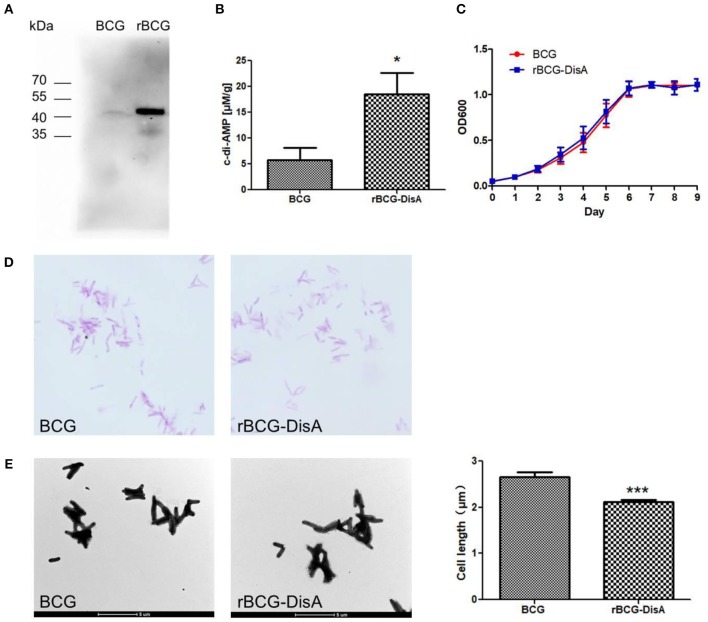
Characterization of rBCG. **(A)** DisA was examined in BCG and rBCG using Western Blot analysis with anti-DisA polyclonal antibody. **(B)** Determination of intracellular c-di-AMP concentrations of the BCG and rBCG-DisA strains by HPLC. **(C)** Bacterial growth curve of the BCG and rBCG-disA strains. The growth was monitored using a microplate reader at 600 nm from day 1 to day 9 at 24-h intervals. **(D)** Morphology of the BCG and rBCG-DisA strains stained with Ziehl-Neelsen acid-fast staining and observed under a light microscope (1 000×). **(E)** Morphology of BCG and rBCG-DisA strains observed by transmission electron microscope (4 200×), and bacterial cell sizes (*n* = 100) were measured by Image *J* software. ^*^P < 0.05, ^***^P < 0.001.

Some bacteria such as *S. aureus* (17), *S. pneumoniae* (39), and *L. monocytogenes* (16), of which has a single diadenylate cyclase. Our previous work had proved that Rv3586 (DisA) is the only diadenylate cyclase in Mtb (20). In BCG, Mb3617 is highly conserved compared to DisA and shows 99.4% identity with DisA amino acids sequence. By transforming a plasmid expressing Mtb *Rv3586* (*disA*) gene, we constructed a recombinant BCG strain, named as rBCG-DisA (Figures S1, S2). The expression of DisA was significantly increased in rBCG-DisA compared to that in BCG using immunoblot with anti-DisA polyclonal antibody (Figure 2A). The levels of c-di-AMP in BCG and rBCG-DisA were determined by HPLC. As predicted, overexpressing *disA* in BCG increased bacterial c-di-AMP levels for 3.2-fold (Figure 2B), indicating that the recombinant DisA is enzymatically active in BCG.

### rBCG With Overexpressed DisA Demonstrated Shorter Bacteria in Size

The diadenylate cyclase is conditionally essential for *L. monocytogenes* when the bacterium is grown in rich media or within host cells (16). *B. subtilis* encodes three diadenylate cyclases, and the triple mutant of cyclases can only grow in low K^+^ media (40). As the only diadenylate cyclase domain protein in Mtb (15, 20), the deletion of Mtb *disA* has little effect on bacterial growth (15). Overexpression of *disA* in BCG did not affect the growth in cultural medium (Figure 2C), as well as the dying by fast-acid staining (Figure 2D). It has been proved that increasing c-di-AMP reduces the bacterial size in CnpB mutant strain of Mtb and Ms (21, 22). In this study, the cell size of rBCG-DisA was 20.4% shorter than wild BCG observed by TEM (Figure 2E). This result indicates that increased c-di-AMP levels lead to the morphologic change of BCG, similar to Mtb, Ms, as well as *S. aureus* (17).

### rBCG-DisA Induces Slightly Stronger Humoral and Cellular Immunity Responses Than BCG

**Figure 3:**
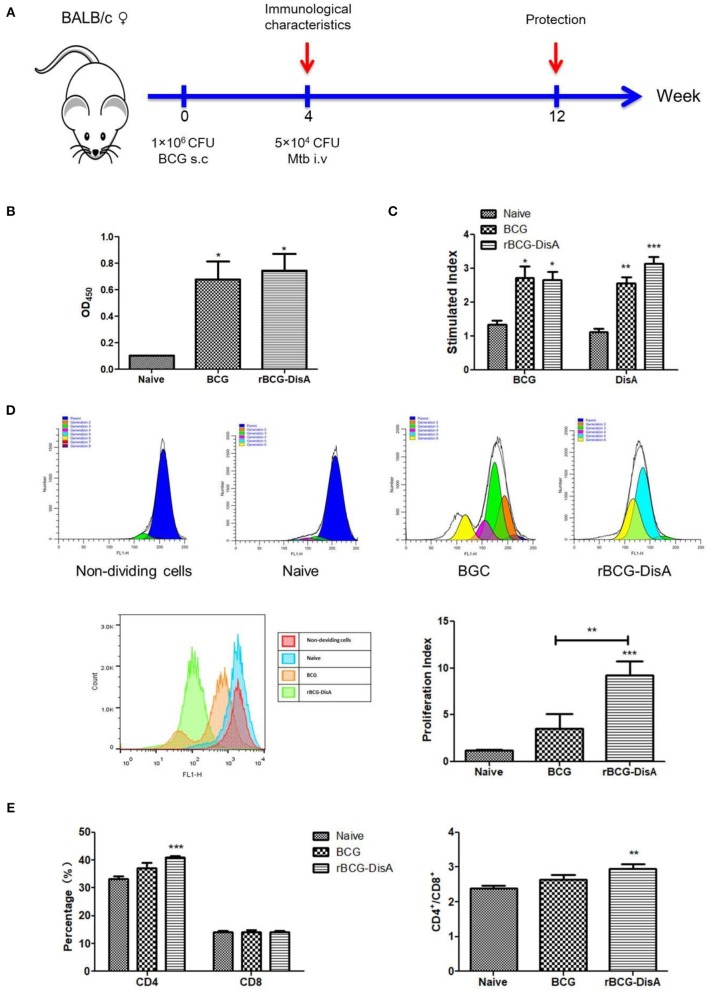
Humoral and cellular immune responses of rBCG-DisA-immunized mice. **(A)** The immunization and Mtb challenging scheme. Female BALB/c mice were subcutaneously (s.c.) immunized with 1 × 10^6^ CFU BCG and rBCG-DisA, respectively, and PBS was used as negative control. At the fourth week after immunization, a group of three mice were sacrificed to determine immunological characteristics. At the same time, three mice were challenged intravenously (i.v) with 5 × 10^4^ CFU virulent Mtb H37Rv strain, and mice injected s.c. with PBS (Naïve) were used as control groups. Eight weeks post-challenge, mice were sacrificed to detect immunological characteristics. **(B)** Four weeks after immunization, sera (1:200) from different groups were collected and total IgG titers of anti-BCG proteins were detected using ELISA. **(C)** Splenic lymphocyte proliferation was assayed using MTS agent after stimulating with BCG and DisA protein, respectively, for 72 h. **(D)** Splenic lymphocyte proliferation was determined using CFSE agent after stimulating with BCG proteins (25 μg/mL) for 7 days, and detected by flow cytometry. **(E)** Percentages of CD4^+^ and CD8^+^ T cells of splenic lymphocytes from each group, and the ratios of CD4/CD8 were calculated as shown. ^*^P < 0.05, ^**^P < 0.01, ^***^P < 0.001.

To further study the rBCG's immunogenicity, we detected the immune response in subcutaneously vaccinated mice with rBCG-DisA as indicated in Figure 3A. Four weeks after immunization, the anti-BCG antibody titer increased significantly in immunized mice compared with the control group, and no difference between BCG and rBCG-DisA groups (Figure 3B).

The proliferation of splenic lymphocyte was determined by MTS assay after BCG and DisA protein stimulation for 72 h. As shown in Figure 3C, cells from BCG and rBCG-DisA immunized mice exhibited significant proliferation responding to BCG protein and DisA protein, and there were no significant differences between rBCG-DisA and BCG groups (*P* > 0.05) (Figure 3C). The results of CFSE assay revealed that the proliferation of splenocytes increased significantly in rBCG-DisA group than the control and BCG groups, but no difference was found between BCG and the control group (Figure 3D). The CD4 T cells proportion showed an increase after immunization in rBCG-DisA group compared with the control group, but no significant difference between rBCG-DisA and BCG groups, BCG, and the control group (*P* > 0.05) (Figure 3E). The proportion of CD8 T cells in each group was similar in profile (Figure 3E). The ratio of CD4/CD8 T cells showed the similar tendency as CD4 T cells proportion in rBCG-DisA-immunized group (Figure 3E).

**Figure 4:**
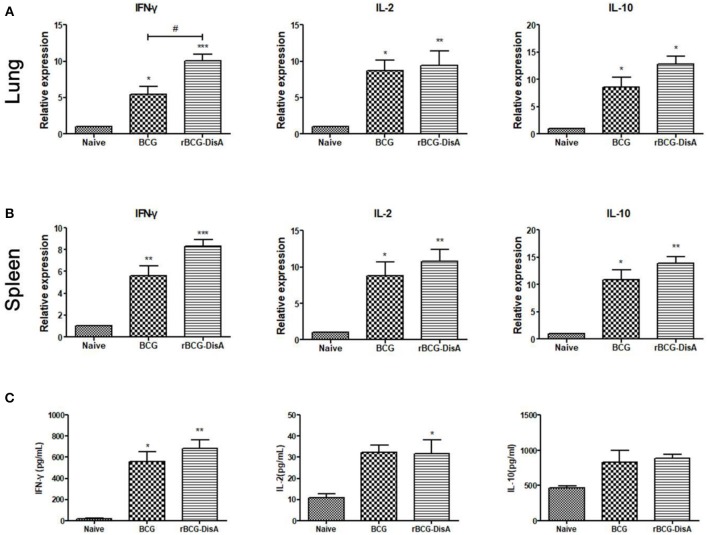
Th1/Th2 cytokine transcription and production in immunized mice. IFN-γ, IL-2 and IL-10 transcription in lungs **(A)** and spleens **(B)** of Naïve, BCG, and rBCG-DisA mice, respectively, were determined by qRT-PCR. Each gene relative expression was normalized by GAPDH. **(C)** Splenocytes from Naïve, BCG-, and rBCG-DisA-immunized mice were stimulated with BCG proteins for 72 h. Culture supernatants were collected and the releases of IFN-γ, IL-2, and IL-10 were analyzed by ELISA. ^*^compared with control group; ^*^^/#^*P* < 0.05, ^**^P < 0.01, ^***^P < 0.001.

Both rBCG-DisA and BCG immunization induced more cytokines transcriptions of IFN-γ, IL-2, and IL-10 in lung and spleen (Figures 4A,B). Significance was found on the expression of Th1 type cytokine IFN-γ between BCG and rBCG-DisA immunized mice in lung, but no significant differences were found on IL-2 and IL-10 between BCG and rBCG-DisA groups both in lung and spleen (Figures 4A,B). Though rBCG-DisA group produced more cytokines than BCG group, no more significant differences were found in cytokines production between the BCG- and rBCG-DisA- immunized groups (Figure 4C). The productions of cytokines by ELISA showed the similar results to that of qRT-PCR. These data indicated that rBCG-DisA induces humoral immune and cellular response as BCG, though some data showed minor increase.

### rBCG-DisA Induced Stronger Cellular Immune Response After Mtb Challenge

**Figure 5:**
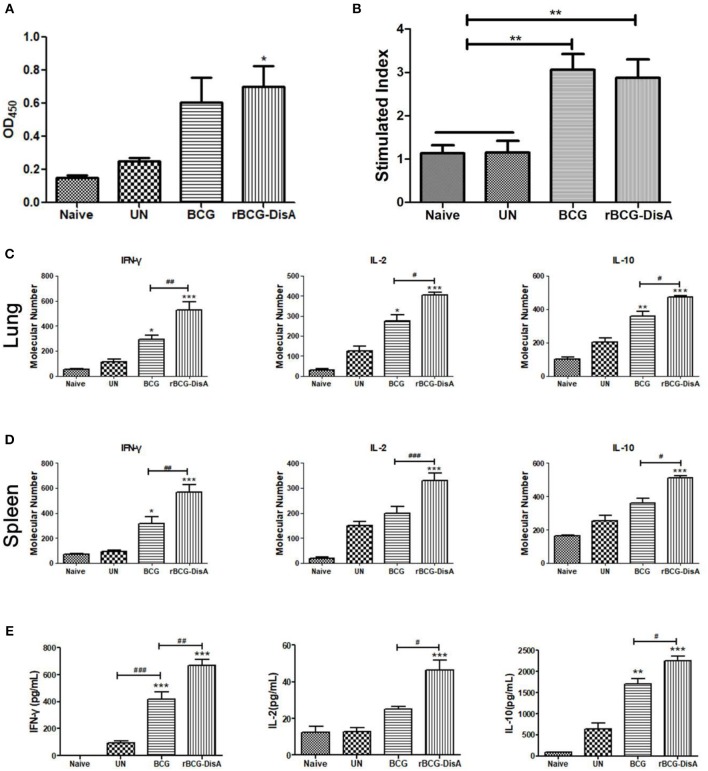
Humoral and cellular immune responses of rBCG-DisA-immunized mice after Mtb infection. **(A)** Eight weeks after Mtb infection, sera from Naïve, un-immunized Mtb-infected mice (UN), BCG-, and rBCG-DisA-immunized mice were collected and total IgG titers of anti-BCG protein were detected by ELISA (1:200). **(B)** Splenic lymphocyte proliferation were assayed by MTS agent after stimulating with BCG protein for 72 h. IFN-γ, IL-2, and IL-10 transcription in lungs **(C)** and spleens **(D)** from Naïve, UN, BCG, and rBCG-DisA-immunized mice were assayed by qRT-PCR. Each gene relative expression was normalized by GAPDH. **(E)** IFN-γ, IL-2, and IL-10 production of splenocytes after stimulation with BCG proteins was measured by ELISA. ^*^compared with control group; ^*^^/#^*P* < 0.05, ^**^^/*##*^*P* < 0.01, ^***^^/*###*^*P* < 0.001.

After 4 weeks of immunization, mice were challenged with Mtb and the immune responses were further characterized 6 weeks post-infection. The antibody titers of rBCG-DisA-immunized mice, comparable with BCG-immunized mice, exhibited at much higher levels than those of the un-immunized mice (Figure 5A). The splenic lymphocytes from BCG- and rBCG-DisA-immunized mice both showed significant proliferation after Mtb challenge than that of the negative and un-immunized mice (Figure 5B). After Mtb infection, spleen lymphocytes from both BCG- and rBCG-DisA-immunized mice produced more cytokines of IFN-γ, IL-2, and IL-10 than infection control. To our surprise, the cytokine levels of rBCG-DisA-immunized mice increased significantly than BCG group, which were much higher than that of vaccination alone (Figure 5E). Similar increasing of Th1/Th2 cytokines was found between BCG and rBCG-DisA-immunized mice in the transcriptions level of IFN-γ, IL-2, and IL-10 (Figures 5C,D). It suggested that rBCG-DisA immunization provided enhanced cellular response after Mtb infection.

### rBCG-DisA Induces Enhanced Innate Immune Responses Than BCG

**Figure 6:**
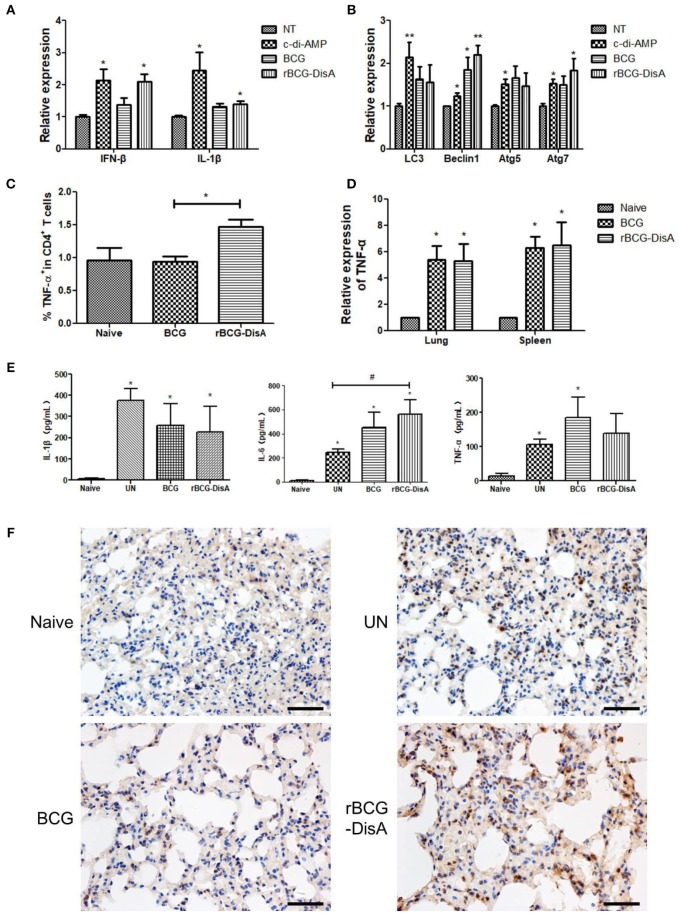
rBCG-DisA induced innate immune responses. **(A)** RAW264.7 macrophages were stimulated with c-di-AMP or infected with indicated bacterial strains at MOI = 10. IFN-β and IL-1β transcripts were analyzed by qRT-PCR. Relative expression of each gene was normalized by expression of GAPDH. **(B)** RAW264.7 macrophages were treated as indicated in **(A)**, and transcription of autophagy related genes (Atgs) including LC3, Beclin1, Atg5, and Atg7 was determined by qRT-PCR. Relative expression of each gene was normalized by expression of GAPDH. **(C)** Splenocytes from Naïve, BCG-, and rBCG-DisA-immunized mice were stimulated with BCG proteins for 72 h. Cells were stained with CD4 and TNF-α fluorescently-labeled antibodies and analyzed by flow cytometry. **(D)** TNF-α transcription in lungs and spleens of Naive, BCG-, and rBCG-DisA immunized mice was measured by qRT-PCR. **(E)** Eight weeks after Mtb infection, splenocytes from Naïve, BCG-, and rBCG-DisA-immunized mice were stimulated with BCG proteins for 72h. IL-1β, IL-6, and TNF-α production was measured by ELISA. **(F)** Eight weeks after Mtb infection, histone 3 trimethylation of lysine 4 (H3K4me3) expressions in lung tissues of Naïve, UN, BCG-, and rBCG-DisA- immunized mice were detected by immunohistochemistry (IHC) and observed by microscope (400×). ^*^^/#^*P* < 0.05, ^**^P < 0.01.

It has been shown that c-di-AMP induces IFN-β release in a STING dependent manner (34), and a robust secretion of IL-1β from macrophages through the NLRP3 inflammasome (30). We predicted that elevated c-di-AMP could enhance the innate immune responses of BCG. In this study, we found that the IFN-β transcription was increased about 2-fold in RAW264.7 macrophages treated with c-di-AMP (Figure 6A). Similar to c-di-AMP, IFN-β transcription significantly increased in rBCG-DisA-infected RAW264.7 macrophage, but there was no significant difference compared with BCG group (Figure 6A). rBCG-DisA infection could induce IL-1β mRNA upregulation as c-di-AMP did compared with BCG group (Figure 6A). It was reported that the IFN-β production was related to autophagy in macrophages infected with Mtb DisA overexpression strain (25). We assayed the transcription of autophagy related genes, such as LC3, Beclin1, Atg5, and Atg7. These genes were up-regulated in macrophages after treatment with c-di-AMP, BCG or rBCG-DisA with un-stimulated cells. rBCG-DisA induced highest levels of Beclin1 and Atg7 expressions among all treatment groups and showed significant differences compared with un-stimulated cells, though no difference between BCG and rBCG-DisA (Figure 6B). Thus, elevated c-di-AMP in rBCG-DisA induced a slight increase of IFN-β response than BCG, and the stimulation of autophagy may contribute to this response.

The splenic lymphocytes from immunized mice were analyzed by flow cytometry, and the results showed that CD4 T cells from the rBCG-DisA-immunized mice produced more TNF-α than those from that of the control group and BCG-immunized group (Figure 6C). The results of qRT-PCR showed that the TNF-α transcription levels in the lung and spleen of the two immunized mice were up-regulated (Figure 6D). After Mtb infection, the secretion of IL-1β, IL-6, and TNF-α of the un-immunized (UN) group and two immunized groups increased compared with the normal control group (Figure 6E). The secretion of IL-6 in the rBCG-DisA-immunized group was significantly increased compared with the UN group (Figure 6E).

Immunohistochemistry (IHC) was used to compare the distributions of epigenetic marker histone 3 trimethylation of lysine 4 (H3K4me3) between different vaccinated mice challenged by Mtb through tail vein injection. It was found that the lungs of normal mouse and challenged mouse without immunization were negative for H3K4 methylation. Strikingly, H3K4 trimethylation revealed obvious positive in two immunized groups, and the rBCG-DisA-immunized mice showed stronger presence of H3K4 trimethylation than the BCG-immunized mice (Figure 6F). Therefore, it is speculated that the enhanced immune response after Mtb infection in rBCG-DisA-immunized mice may be related to innate immune memory, also known as trained immunity.

### rBCG-DisA Provided Protection Efficiency Comparable to BCG Against Mtb Challenge

**Figure 7:**
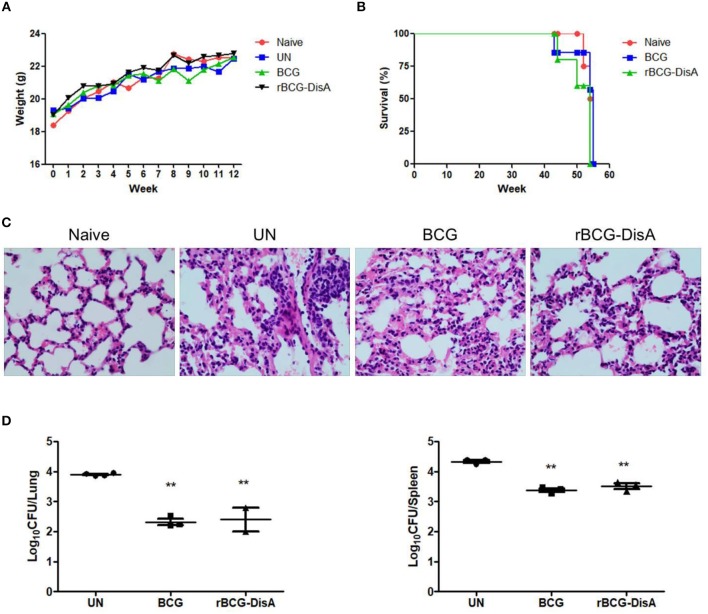
Evaluation of immunoprotection of rBCG-DisA in Mtb-infected mouse model. **(A)** Body weight changes of mice in each group during the whole experiment. **(B)** Survival rates of Naïve mice, as well as BCG- and rBCG-DisA-immunized mice. All group mice were naturally dead after 43–55 week immunization. **(C)** HE-stained lung sections of Naive, UN, BCG-, and rBCG-DisA- immunized mice at 8 weeks post Mtb infection. Gross pathology changes were observed by microscope (400×). **(D)** After 8 weeks of Mtb infection, bacterial burdens in the lungs and spleens of UN, BCG- and rBCG-DisA-immunized mice, respectively, were determined by plate CFU counting. ^**^P < 0.01.

Mtb strains with increased c-di-AMP levels, either overexpressing DisA (25) or absence of PDE (CnpB) (15), showed attenuation in infected mice than WT Mtb. Herein, our data showed that appetite, activities and body weight changes of BCG- and rBCG-immunized mice after Mtb infection were similar to those in normal mice, and the body weight of all groups showed an increasing trend (Figure 7A). After Mtb infection, all infected mice showed similar pathological manifestations of chronic inflammation (Figure 7C). As shown in Figure 7C, tubercular characterized nodules and granuloma were not found in all slides from infected mice. The alveolar structures were intact, with occasional thickening of alveolar mesenchyme, inflammatory cell infiltration, erythrocyte and histological fluid exudation (Figure 7C). Thus, the pathological change of rBCG-DisA-immunized mice was similar to that of BCG-immunized mice. The bacterial burdens in the lungs and spleens of BCG- and rBCG-DisA-immunized mice were significantly decreased compared with the un-immunized group (UN group), and there was no significant difference between two immunized groups (Figure 7D). In addition, BCG- and rBCG-DisA-immunized mice were naturally dead as normal mice after 43–55 week immunization (Figure 7B). Expression of DisA in BCG does not show visible side effects after Mtb infection in mice.

## Discussion

In our previous work, we found that the expression of Mtb and BCG DisA were very low when detected with polycolonal antibody against recombinant DisA (36). In this study, specific anti-DisA antibody titers in sera from TB patients and Mtb-infected animals were also very low (Figures 1B,C), supporting the previous results that c-di-AMP was hard to be detected in Mtb and BCG by either HPLC (25) or ELISA (15). The overexpression of *disA* in BCG had brought about 3-fold increase in c-di-AMP content (Figure 2B). Several reports showed that c-di-AMP regulates the growth and cell size, such as *S. pneumonia* (18)*, L. monocytogenes* (16), *Streptococcus suis* (41), and *S. aureus* (17). The deletion of c-di-AMP hydrolase in Mtb led to the bacterial c-di-AMP accumulation and the bacterial length was reduced (15). Similarly, the bacterial size of rBCG-DisA was shorter than wild type strain (Figure 2E), consistent with the Mtb *cnpB* mutant strain with elevated c-di-AMP (15). Overexpression of c-di-AMP synthetase in Ms produced small colonies with a convex and smooth appearance (22). However, there were no apparent phenotypic changes in growth rate (Figure 2C) as well as the colony morphology (Data not shown) between rBCG-DisA and the wild type BCG strain.

c-di-AMP was used as mucosal adjuvants to enhance the immunogenicity of subunit vaccines (31). Previously, we discovered that DisA of Mtb held potent immunogenicity and elicited high humoral response in mice (36). Our results showed that rBCG-DisA induced slightly higher humoral and cellular responses (Figures 3, 4). These results suggested that the better immune response benefit mainly from the increase of endogenous c-di-AMP accumulation but less from the *per se* immunogenicity of DisA. Surprisingly, either low or high c-di-AMP levels led to accumulation of ppGpp in *L. monocytogenes* as well as *S. aureus*, causing a metabolic disturbance and possibly presenting an alert signal for stringent responses (42–44). There might be an elusive regulatory mechanism existing in rBCG-DisA that prevents moderate levels of c-di-AMP from being too high or too low to avoid possible physiological disturbances within the bacteria, which may explain that the level of immune response is not significantly increased by rBCG-DisA compared with BCG.

As an intracellular bacterium, Mtb primarily survives and multiplies in macrophages after infection, and the cellular immune response mediated by T cells is crucial and dominates the elimination process of Mtb (45–48). Helper T (Th) cells produce cytokines, especially Th1 type cytokines, which are thought to play a major protective role in anti-Mtb infection (49). MTS and CFSE assay results showed that rBCG-DisA produced stronger lymphocytes proliferation except for CD8 T cells proliferation in the mouse spleens than BCG (Figures 3C–E). Instead, rBCG-DisA in mice induced an increased expression of Th1 type cytokines such as IFN-γ and IL-2 (Figures 4A,B), and similar scenarios were also detected in the splenic lymphocytes with particularly high secretion of IFN-γ, though there was not statistic difference between the BCG- and rBCG-DisA-immunized groups (Figure 4C). However, the percentages of CD4 T cells, capable of secreting IFN-γ and/or IL-2, were increased by rBCG-DisA compared to that induced by BCG (Figure S3), suggesting that the stronger cellular immune response induced by rBCG-DisA may be likely to be attributed to the increase of CD4 T cells. After Mtb infection, cellular immune responses were fairly detected as the significant proliferation of spleen lymphocytes was present, yet the rBCG-DisA-immunized group was not statistically higher than the BCG-immunized group (Figure 5B). However, it was still noteworthy that the transcription and secretion of cytokines in spleens and lungs in the rBCG-DisA group were significantly higher than those in the BCG and UN groups (Figures 5C–E), suggesting that rBCG-DisA induces stronger cellular immunity than BCG. In BCG group, the secretion of IL-10 after Mtb infection (Figure 5E) was higher than BCG immunization (Figure 4C), and IL-2 of BCG immunization mice (Figure 4C) was similar to that challenged with Mtb (Figure 5E) but was not higher than the normal group. Whereas, the rBCG-DisA-induced cytokine response was significantly increased after Mtb infection (Figures 5C–E), suggesting that rBCG-DisA may exist another mechanism different from BCG to induce more potent cellular immune responses.

c-di-AMP induces an innate immune response characterized by elevated IFN-β and is considered to be a key pathogen associated molecular pattern (PAMP) (25). Our study showed that rBCG-DisA-infected RAW264.7 macrophages induced higher IFN-β transcription than un-stimulated cells, but not BCG-infected cells (Figure 6A). rBCG-DisA activated Atgs increase as Beclin1 and Atg7 transcription augmented (Figure 6B), but the overall autophagy did not increase significantly (Figure S4), which was inconsistent with a previous paper reporting that Mtb with overexpressed DisA induced potent autophagy (25). It is unclear how c-di-AMP is secreted outside BCG, but there is evidence that both the secretion of c-di-AMP and RD1 (region of difference 1 (RD1) between Mtb and BCG) are required for c-di-AMP-mediated innate immunity response (34). However, c-di-AMP secretion in BCG is defective and it is not because of lacking ESX-1 (34), whereas the direct introduction of c-di-AMP into rBCG-DisA did not produce an elevated autophagy in macrophages as strong as Mtb did, simply indicating that BCG differs from Mtb in c-di-AMP secretion machinery.

Trained immunity is defined as a heightened response to a secondary infection that can be exerted both toward the same microorganism and a different one (50). The activation innate immune memory was marked by an increase in H3K4 histone modification (51), followed with releases of cytokines such as IL-1β, TNF-α, and IL-6 (52, 53). Here, we showed that increased IL-1β response induced by c-di-AMP and rBCG-DisA infection in RAW264.7 macrophages, but not in BCG-infected cells (Figure 6A). Though the total transcription and release of TNF-α were not increased significantly, the percentage of TNF-α production cells in CD4^+^ T cell from rBCG-DisA-immunized mice was much higher than the BCG-immunized group (Figures 6C,D). In addition, the H3K4 histone modification (the characteristic of innate immune memory) in the rBCG-DisA-immunized mice was significantly increased (Figure 6F). These results strongly suggest that the increased immune response induced by rBCG-DisA after Mtb infection may be associated with an enhanced trained immunity because of the elevated c-di-AMP in rBCG-DisA, since it is well-known that innate immunity determines the strength and type of adaptive immunity.

With the emergence of global multidrug-resistant Mtb, HIV, and TB co-infected immunocompromised patient's vaccination need to be carefully considered. The safety of new vaccines is a priority issue. The attenuation of virulence in Mtb Δ*cnpB* mutant strain was due to elevated c-di-AMP because of the same phenotype obtained with DisA-overexpressing strain (15, 25, 33). Elevated c-di-AMP in BCG enhanced the innate and adaptive immunity in mice, especially after Mtb re-infection. It is inspirited that no adverse reactions were observed in experimental animals including weight and survival after rBCG-DisA immunization (Figures 7A,B) as well as the pathological changes of lungs in two immunized groups were comparable after Mtb infection (Figure 7C). Based on these reports and our findings, we speculate that rBCG-DisA may be safer than wild type BCG. However, further study needed to be explored with immunodeficient mice to assess the safety of rBCG-DisA comprehensively.

Though rBCG-DisA immunization induced stronger immune response, especially after Mtb infection, its protection against Mtb infection was not superior to wild type BCG. Despite of insufficient efficiency, BCG is considered as a successful and most widely used vaccine for TB, and also a strong immune enhancer as the main ingredient of Freund's adjuvant. Elevated c-di-AMP in rBCG-DisA had changed the immunogenicity of BCG from our results, but the enhancement may be not sufficient to be distinguished with the immune responses induced by BCG alone. The Mtb infection model could be established by intravenous (54–56), aerosol (25, 48), or intranasal (47, 57) infection routes. In this study, we used intravenous infection of mice as a model for evaluation study, which resulted in much higher bacteria loads than human tuberculosis. There comes the possibility that the immunization enhancement of c-di-AMP may be masked. So more susceptible animals, such as guinea pigs, and aerosol infection should be used for evaluation of rBCG-DisA against Mtb in the future. Other than this, the finding of trained immunity reveals BCG versatile on non-mycobacterial prevention, even tumor treatment. Our important finding proved that rBCG-DisA may induce stronger trained immunity. It is worth to explore multiple application potentials of rBCG-DisA on other infectious diseases as well as tumor biotherapy.

Taken together, our results indicated that the elevation of c-di-AMP in BCG enhances the immunogenicity of BCG and thus induces a stronger immune response both in macrophages and immunized mice, especially after Mtb infection, which is related with trained immunity. Thus, rBCG-DisA and c-di-AMP as promising immunomodulatories should be further assessed for multiple application potentials in TB, other infectious diseases and even cancer therapy.

## Conclusion and Perspective

Tuberculosis remains one of the most deadly killers of infectious diseases worldwide which is caused by *Mycobacterium tuberculosis*. Bacillus Calmette-Guerin (BCG) is a live attenuated vaccine against tuberculosis and remains the most commonly used vaccine worldwide. However, BCG has varied protective efficacy, and effective vaccines are necessary for preventing the prevalence of tuberculosis. Cyclic di-AMP is a bacterial second messenger which regulates various cellular processes and host immune response and is considered to be a key pathogen associated molecular pattern. Here recombinant BCG with elevated cyclic di-AMP is constructed by overexpressing diadenylate cyclase of *Mycobacterium tuberculosis* in BCG. Recombinant BCG elevated cyclic di-AMP exhibits the similar growth to BCG but shorter bacterial cell size. Recombinant BCG immunization induces similar humoral and cellar immune responses with BCG in a mouse vaccination model. Cyclic di-AMP produced by the recombinant BCG served as endogenous adjuvant and rendered stronger cellular immune responses and certain protection after *Mycobacterium tuberculosis* challenge. Hence, rBCG-DisA and c-di-AMP as promising immunomodulatories should be further assessed for multiple application potentials in TB, other infectious diseases and even cancer therapy.

## Data Availability

The raw data supporting the conclusions of this manuscript will be made available by the authors, without undue reservation, to any qualified researcher.

## Author Contributions

HN, LW, JZ, YL, and JK performed the experiments. HN, LW, JZ, YL, TD, and LS analyzed the data. HN and YB wrote the manuscript. LW and YB conceived and designed the research. YB and ZX supervised this work. All of the authors have read and agreed with the data.

### Conflict of Interest Statement

The authors declare that the research was conducted in the absence of any commercial or financial relationships that could be construed as a potential conflict of interest.
