# Supplementary material for: Recombinant BCG With Bacterial Signaling Molecule Cyclic di-AMP as Endogenous Adjuvant Induces Elevated Immune Responses After Mycobacterium tuberculosis Infection
Source: Front Immunol. 2019 Jul 3;10:1519. doi: 10.3389/fimmu.2019.01519 (PMC6618344; doi:10.3389/fimmu.2019.01519)
Supplement: Supplementary file 1 [file Data_Sheet_1.doc]

**Table S1 Primers used in this study**

| **Primer** | **Sequence (5’ to 3’)** *^a^* | **Purpose** |
| --- | --- | --- |
| **Recombinant strain construction** | | |
| Pr95 | TTTAAGCTTATGCACGCTGTGACTCGTCCGACC | *disA* upstream, forward |
| Pr111 | GCGAAGCTTAAGGCGGATAATTATTGATCGC | *disA* downstream, reverse |
| Pr115 | TTTGTACCGCTCCAGGGTTG | *disA* internal, forward |
| Pr117 | ATCGCTGATGGTCGATTCCG | *disA* internal, reverse |
| Pr120 | GACCGCGGCATAACTGAAAAC | upstream of the MCS of PW54 |
| **qRT-PCR** | | |
| Pr28 | ACAACTTTGGCATTGTGGAA | mouse *gapdh*, forward |
| Pr29 | GATGCAGGGATGATGTTCTG | mouse *gapdh*, reverse |
| Pr32 | CATCTTCTCAAAATTCGAGTGACAA | mouse *tnf-α*, forward |
| Pr33 | TGGGAGTAGACAAGGTACAACCC | mouse *tnf-α*, reverse |
| Pr58 | GCTCTGAGACAATGAACGCTAC | mouse *ifn-γ*, forward |
| Pr59 | TGCAGGATTTTCATGTCACC | mouse *ifn-γ*, reverse |
| Pr60 | CTTGTGCTCCTTGTCAACAG | mouse *il-2*, forward |
| Pr61 | TCCAAGTTCATCTTCTAGGC | mouse *il-2*, reverse |
| Pr64 | TGCTCTTACTGACTGGCAT | mouse *il-10*, forward |
| Pr65 | CTGGATCATTTCCGATAAGGC | mouse *il-10*, reverse |
| Pr257 | GTCCACGCTCGACCTTCTTAC | mouse *beclin1*, forward |
| Pr258 | CACTTGCCAGTCTTAACCTCTG | mouse *beclin1*, reverse |
| Pr259 | TGTGCTTCGAGATGTGTGGTT | mouse *atg5*, forward |
| Pr260 | ACCAACGTCAAATAGCTGACTC | mouse *atg5*, reverse |
| Pr261 | TCTGGGAAGCCATAAAGTCAGG | mouse *atg7*, forward |
| Pr262 | GCGAAGGTCAGGAGCAGAA | mouse *atg7*, reverse |
| Pr263 | TTATAGAGCGATACAAGGGGGAG | mouse *lc3*, forward |
| Pr264 | CGCCGTCTGATTATCTTGATGAG | mouse *lc3*, reverse |
| Pr273 | GAAATGCCACCTTTTGACAGTG | mouse *il-1β*, forward |
| Pr274 | TGGATGCTCTCATCAGGACAG | mouse *il-1β*, reverse |
| Pr275 | AGCTCCAAGAAAGGACGAACA | mouse *ifn-β*, forward |
| Pr276 | GCCCTGTAGGTGAGGTTGAT | mouse *ifn-β*, reverse |

*a* The restriction site is underlined if presents in sequence.


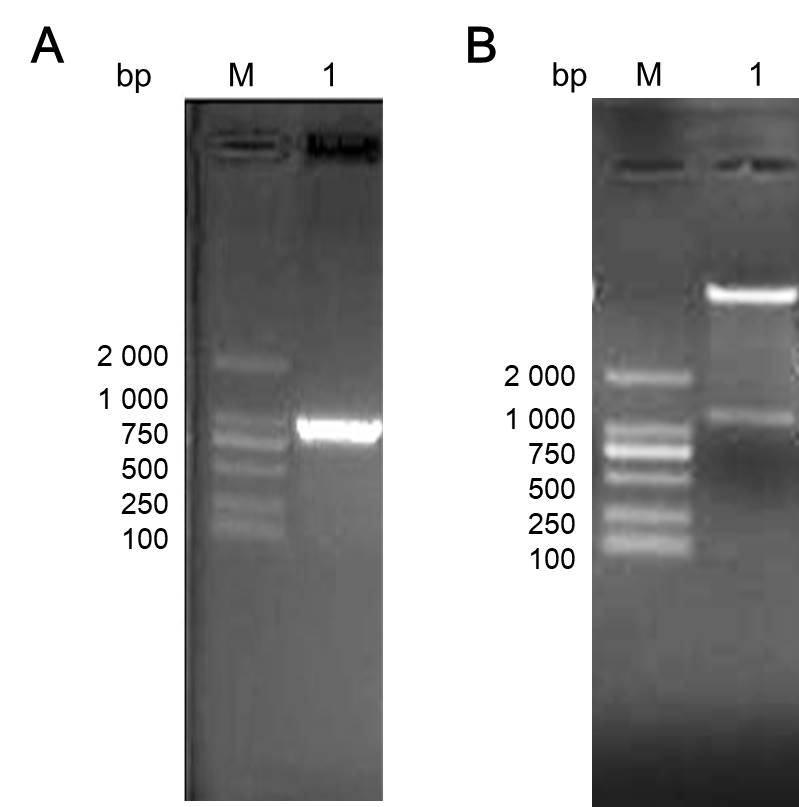


**FIGURE S1 Amplification of *Rv3586* (*disA*) and identification of recombinant plasmid**

(A) With Pr95 and Pr111, *Rv3586* (*disA*) gene fragment was amplified by PCR using Mtb H37Rv genome as template, and PCR products were observed by 1% agarose gel.(B) The fragment of *disA* and *E.coli*-mycobacterium shuttle vector PW54 were digested by *Hin*d III restriction endonuclease, and fragments were observed by 1% agarose gel.


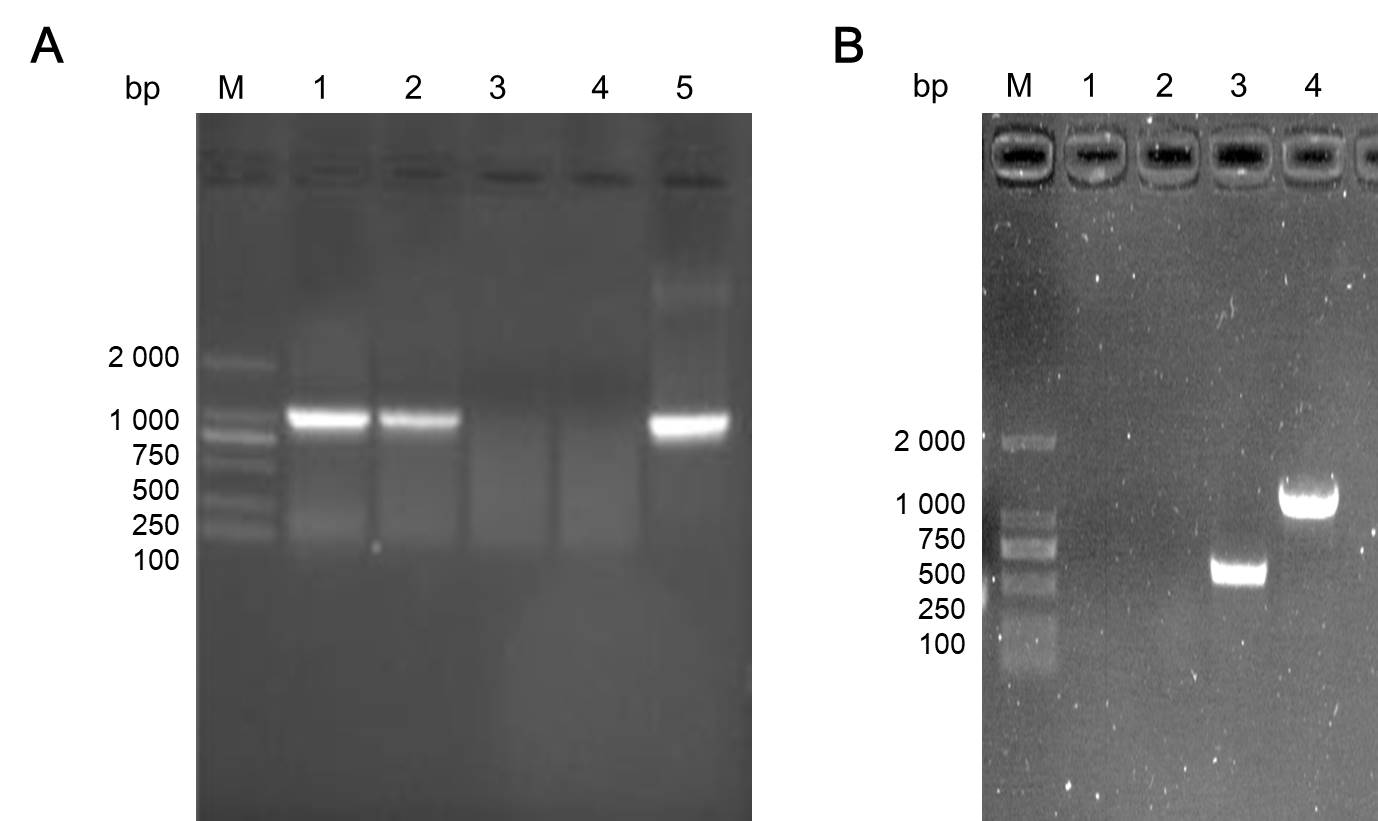


**FIGURE S2 Identification of rBCG transformants and genotypes**

(A) Recombinant BCG (rBCG) transformants were identified by PCR with Pr120 and Pr111, and PCR products were observed by 1% agarose gel. (B) Genotypes of No.1 transformmant in (A) were identified by PCR, and PCR products were observed by 1% agarose gel. Lane 1, negative control with BCG (Pr120 and Pr115); Lane 2, negative control with BCG (Pr120 and Pr117); Lane 3, PCR product of rBCG (Pr120 and Pr115); Lane 4, PCR product of rBCG (Pr120 and Pr117).


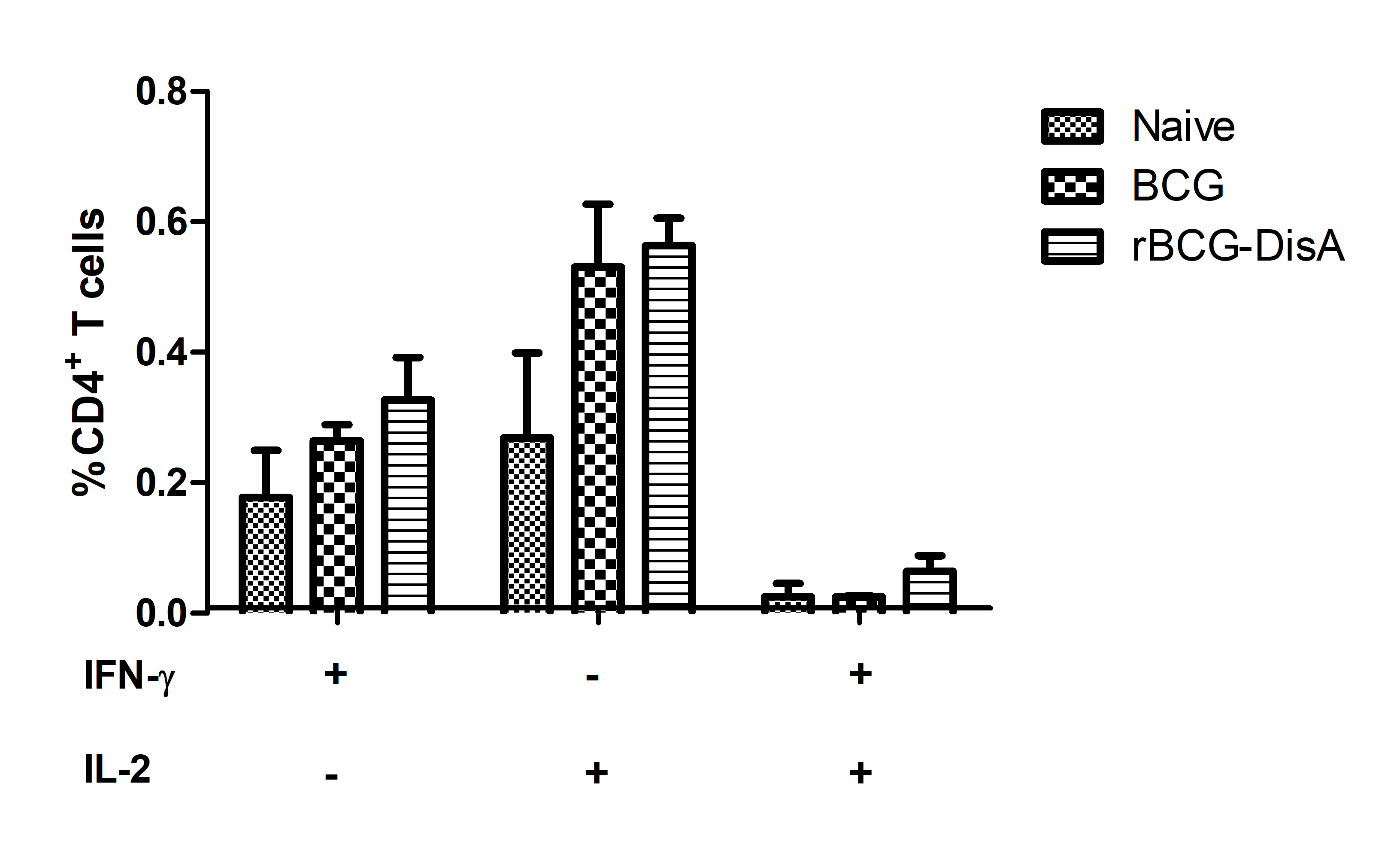


**FIGURE S3 The expressions of IFN-γ and IL-2 in immunized mice detected by flow cytometry**

At the fourth week after immunization, splenic lymphocytes were stimulated with BCG protein (25μg/mL) for 72 hours. At the last 12 hours of stimulation, BrefedlinA was added. Intracellular cytokines including IFN-γ and IL-2 labelling was performed after surface CD4 staining. The percentages of indicated cells were analyzed by flow cytometry.


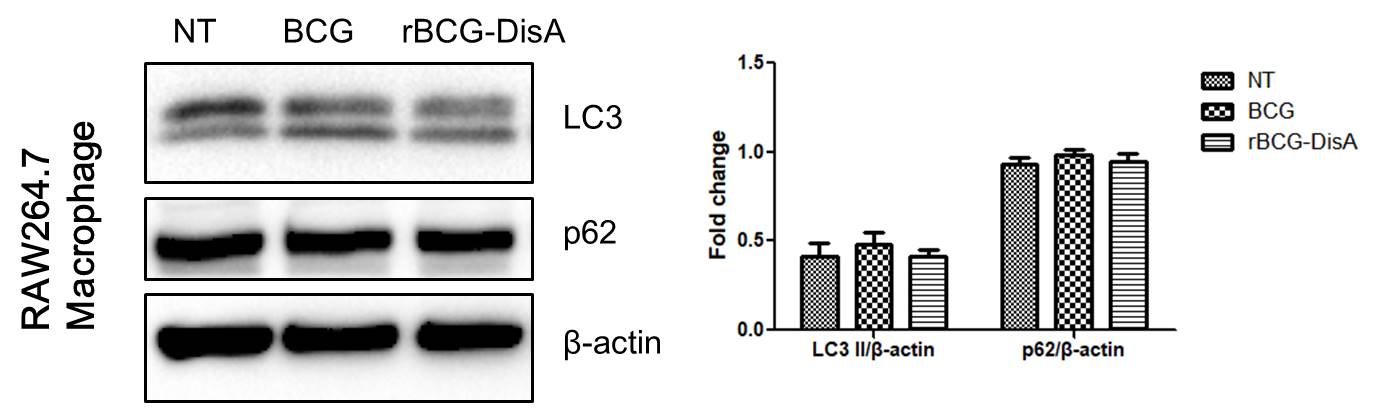


**FIGURE S4 LC3 and p62 expressions in RAW264.7 macrophages after BCG infection**

RAW264.7 cells were infected with BCG and rBCG-DisA at MOI = 10. After 24 hours infection, cellular proteins were extracted used RIPA lysate containing protease inhibitor on ice. The expressions of LC3 and p62 were analyzed by Western blot, and relative expression were standardized by β-actin.
